# Prostate cancer grade migration and facility-level treatment trends for grade group 1 disease

**DOI:** 10.1093/jncics/pkad018

**Published:** 2023-02-25

**Authors:** Leonardo D Borregales, Michael Tzeng, Ashwin Ramaswamy, Xiangmei Gu, Meenakshi Davuluri, Himanshu Nagar, Jim C Hu

**Affiliations:** Department of Urology, Weill Cornell Medicine/New York-Presbyterian, New York, NY, USA; Department of Urology, Weill Cornell Medicine/New York-Presbyterian, New York, NY, USA; Department of Urology, Weill Cornell Medicine/New York-Presbyterian, New York, NY, USA; Department of Healthcare Policy and Research, Weill Cornell Medicine, New York, NY, USA; Department of Urology, Weill Cornell Medicine/New York-Presbyterian, New York, NY, USA; Department of Radiation Oncology, Weill Cornell Medicine/New York-Presbyterian, New York, NY, USA; Department of Urology, Weill Cornell Medicine/New York-Presbyterian, New York, NY, USA

## Abstract

Overdiagnosis and overtreatment of low-grade prostate cancer (PCa) reflect poor quality of care and prompted changes to guidelines over the past decade. We used the National Cancer Database to characterize Gleason Grade Group (GG)1 PCa diagnosis trends and assess facility-level treatment variability. Between 2010 and 2019, GG1 PCa incidence had a clinically and statistically significant decline, from 45% to 25% at biopsy and from 33% to 9.8% at radical prostatectomy (RP) pathology. Similarly, active surveillance (AS) uptake significantly increased to 49% and 62% among nonacademic and academic sites, respectively. Decreasing rates of definitive therapies were identified: among academic sites, RP decreased from 61.1% to 25.3% and radiation therapy (RT) from 25.2% to 12%, whereas among nonacademic sites, RP decreased from 53.6% to 28% and RT from 37.8% to 21.9% (*P*_trend_ < .001). Declines in the diagnosis and treatment of low-grade disease demonstrate an encouraging shift in PCa epidemiology. However, heterogeneity in AS utilization remains and reflects opportunities for improvement.

A significant challenge to prostate cancer (PCa) screening is maximizing the detection of aggressive disease while minimizing overdiagnosis of low-grade disease. To avoid overdiagnosis, professional guidelines advocate a risk-stratified approach, limiting the age-group recommended screening (55-69 years) and recognizing the use of prostate magnetic resonance imaging and biomarkers to inform the decision to pursue prostate biopsy (1,2). Encouragingly, early indications suggest adherence to contemporary guidelines as evidenced by decreasing prevalence of Gleason grade group (GG)1 disease detected on radical prostatectomy (RP) pathology in the United States and abroad (3,4,5).

To avoid overtreatment, active surveillance (AS) is the preferred choice for low-grade PCa. This is strongly endorsed because of excellent oncological safety and ability to mitigate the consequences of definitive treatment with surgery or radiation (1). Nonetheless, despite increased support for AS (6), uptake in the United States continues to lag behind other health-care systems, such as Sweden, where AS utilization for GG1 disease reached 74% by 2014 (7). Lack of AS adoption has been attributed to a statistically significant degree of variation and heterogeneity in facility-level practice patterns in the United States; purportedly, academic hospitals advocate AS more strongly for low-grade PCa than nonacademic centers (8). Contemporary trends in GG1 PCa incidence and management can inform providers and policy makers on the current state of AS utilization and guide future initiatives. Therefore, using a nationally representative cancer registry, we sought to characterize trends in the incidence of GG1 disease on prostate biopsy and RP pathology and assess treatment variability by facility type.

Men with newly diagnosed PCa between 2010 and 2019 were identified in the National Cancer Database (NCDB), a Commission on Cancer–accredited hospitals registry in the United States (9). Analysis of trends in GG distribution included 1 024 607 men in the biopsy cohort and 511 199 men in the RP cohort. Analysis of treatment trends (AS vs RP, radiation therapy [RT], local ablation, or androgen deprivation therapy) of localized GG1 PCa according to facility type included 249 585 men. Patients with unknown stage and/or grade, treatment, or facility type were excluded. The selection of men for the 3 study cohorts is illustrated in Supplementary Figures 1 and 2 (available online). Facility type was defined as academic (“academic/research program”) vs nonacademic (“community cancer program”, “comprehensive community cancer program”, or “integrated network cancer program”). The NCDB explicitly defines active surveillance as code (2) in the variable “RX_SUMM_TREATMENT_STATUS”, and men who do not receive any primary therapy are not considered to have undergone AS. We followed this methodology to abstract only patients who underwent initial AS for management of their PCa, as previously validated by Loppenberg et al. (8).

Changes in the distribution of GG1 for biopsies and RP pathology between 2010 and 2019 were assessed using the Cochran-Armitage test for trend. Joinpoint Regression Program 4.9.0.1 was used for joinpoint analysis. The regression model was fit to calculate the regression coefficient, average annual percent change (AAPC), and corresponding 95% confidence intervals (CIs). The level of statistical significance was defined as a *P* value less than .05. All analyses were performed using SAS (SAS Institute, Cary, NC, USA).

Between 2010 and 2019, the proportion of GG1 detected on biopsy decreased from 44.9% to 24.6% (*P* < .001), and the proportion of GG1 detected on RP decreased from 33.0% in 2010 to 9.8% in 2019 (*P* < .001) (Figure 1). Sociodemographic and clinical characteristics are shown in Supplementary Table 1 (available online). Within academic sites, the use of AS increased from 12.1% to 61.8% (AAPC = 20.5%, 95% CI = 18.6 to 22.4), RP decreased from 61.1% to 25.3% (AAPC = -9.3%, 95% CI = -10.2 to -8.5), and RT decreased from 25.2% to 12% (AAPC = -7.9%, 95% CI = -8.8 to -7.0) (Figure 2). Similar trends were seen in nonacademic sites, where the use of AS increased from 5.4% to 48.6% (AAPC = 27.8%, 95% CI = 24.1 to 31.6), RP decreased from 53.6% to 28% (AAPC = -6.9%, 95% CI = -8.7 to -5.2), and RT decreased from 37.8% to 21.9% (AAPC = -5.9%, 95% CI = -6.6 to -5.3; *P*_trend_ < .001). Androgen deprivation therapy and local ablation were scarcely used among both facility types, with less than 1% of patients receiving such therapies by 2019 (Supplementary Tables 2 and 3, available online).

**Figure 1.**
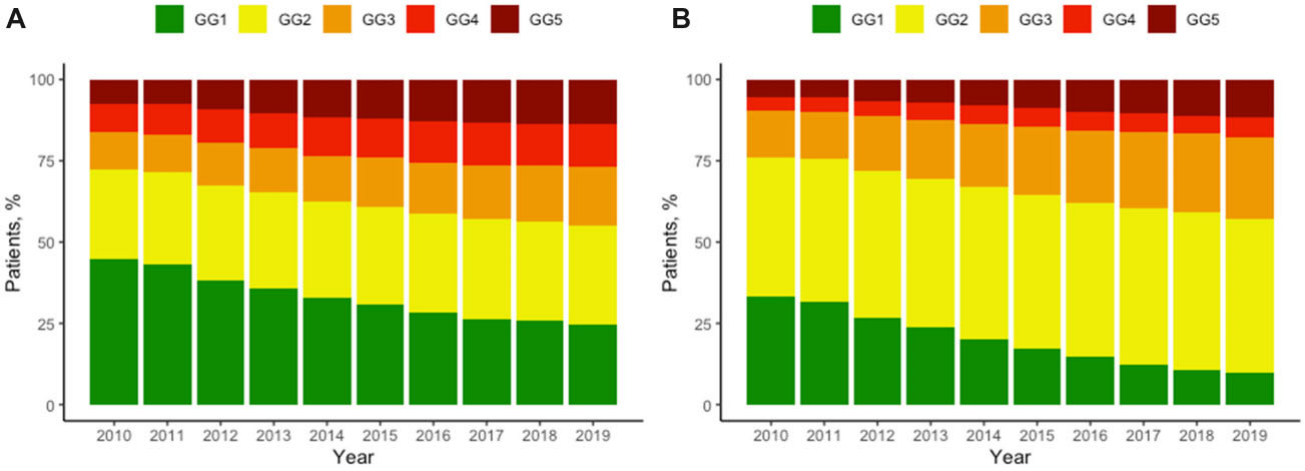
Distribution of Gleason grade group (GG) 1-5 on **A)** biopsy and **B)** radical prostatectomy pathology, National Cancer Database 2010-2019.

**Figure 2.**
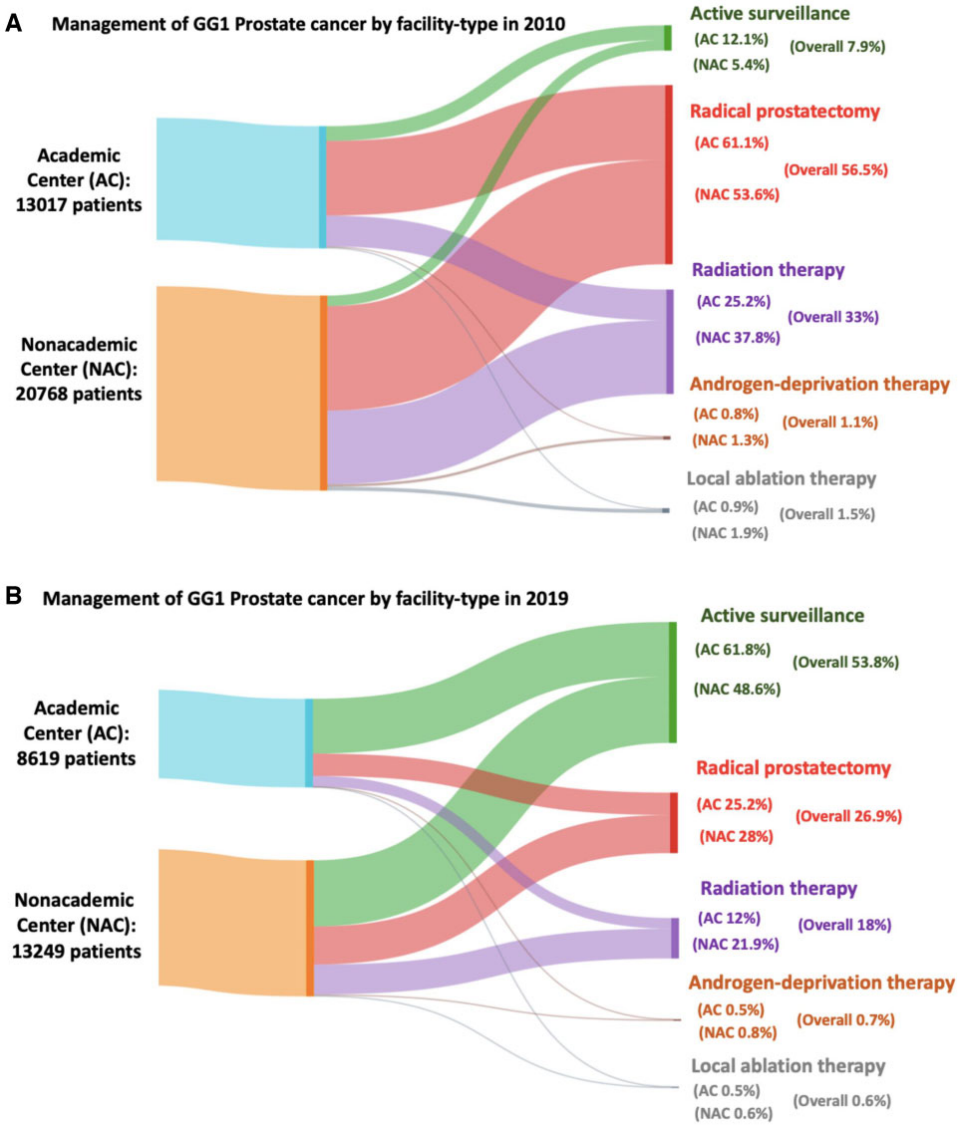
Management of GG1 prostate cancer by facility type. Flow diagram depicts the relative proportion of men in **A)** 2010 and **B)** 2019 with GG1 prostate cancer managed at academic (**turquoise**) or nonacademic centers (**orange**) and corresponding proportions who were subject to active surveillance (**green**), radical prostatectomy (**red**), radiation therapy (**purple**), androgen-deprivation therapy (**brown**), and local ablation therapy (**silver**). The thickness of each colored curve corresponds to the relative proportion for the respective originating node. GG = Gleason grade group.

Two key findings are highlighted in our analysis. Firstly, we demonstrated a clinically significant decline in the incidence of GG1 PCa cases detected (Supplementary Table 4, available online) and treated over the last decade. GG1 is no longer the most commonly diagnosed grade, reflecting a significant epidemiologic shift in PCa. Moreover, a decline in the utilization of definitive therapies was identified in academic and nonacademic sites, suggesting improvements in the proper stewardship of PCa screening and treatment.

Previous NCDB studies identified stage and grade migration phenomena through 2014 (10). Our study validates the increasing proportion of high-grade disease detected on biopsy with contemporary data, and it is the first to report a similar trend in RP pathology with GG1 disease in this cohort. Namely, we found that by 2019 only 9.8% of RP specimens harbored GG1 disease, which parallels European studies (3,4). This trend reflects improved discrimination for diagnosing low-grade disease, possibly because of changes in screening practices and a restrictive approach to biopsy (5).

The uptake of AS in nonacademic settings has been previously described. A study of community-based practices in Michigan found that 49% of men with low-risk disease underwent AS in 2012-2013 (11). Similarly, 48% of low-risk men in the Veterans Affairs–integrated health-care system were managed conservatively between 2005 and 2015 (12). In this study, AS utilization for GG1 PCa increased dramatically across practice settings; however, among nonacademic centers, the majority of men continued to receive definitive treatment by 2019. Although 62% of men received AS at academic sites, the United States lags behind other developed countries in adopting guideline-concordant care for GG1 PCa, representing an opportunity for improvement. A limitation of our study is that data are derived from a US hospital-based registry; hence, the interpretation of the trends here is limited to Commission on Cancer–designated facilities. Additionally, many men were excluded because of unknown treatment or missing data; therefore, the study is prone to selection bias. However, the strength of this study is the use of nationwide quality data, which differentiates AS as a unique treatment strategy, contrary to other population-based registries.

In conclusion, we found decreasing rates of GG1 detected on biopsy and RP alongside increased use of AS in academic and nonacademic institutions; however, most men with GG1 disease at nonacademic sites received definitive treatment in 2019, reflecting an opportunity for improvement.

## Supplementary Material

pkad018_Supplementary_Data

## Data Availability

The data underlying this article were provided by the American College of Surgeon Commission on Cancer by permission granted to Dr Nagar. Data will be shared on request to the corresponding author with permission of the American College of Surgeon Commission on Cancer.
