# Supplementary material for: Prostate cancer grade migration and facility-level treatment trends for grade group 1 disease
Source: JNCI Cancer Spectr. 2023 Feb 25;7(2):pkad018. doi: 10.1093/jncics/pkad018 (PMC10133398; doi:10.1093/jncics/pkad018)

Supplementary Table 1. Descriptive characteristics for patients with localized Gleason grade 1 prostate cancer by treating facility type.

	**Non-academic**	**Academic**
Patients, n	147,573	102,012
Age, years, median (IQR)	63 (58-68)	62 (57-67)
PSA, ng/mL, median (IQR)	5.6 (4.4-7.9)	5.4(4.3-7.5)
Year of diagnosis, n (%)		
2010	20,768 (14.1)	13,017 (12.8)
2011	20,614 (14.0)	13,738 (13.5)
2012	14,857 (10.1)	10,303 (10.1)
2013	13,784 (9.3)	9,801 (9.6)
2014	12,478 (8.5)	8,718 (8.6)
2015	12,521 (8.5)	9,326 (9.1)
2016	13,031 (8.8)	9,549 (9.4)
2017	13,342 (9.0)	9,725 (9.5)
2018	12,929 (8.8)	9,216 (9.0)
2019	13,249 (9.0)	8,619 (8.5)
Race, n (%)		
White	118,220 (80.1)	75,585 (74.1)
Black	19,367 (13.1)	15,629 (15.3)
Hispanic	5,521 (3.7)	5,843 (5.7)
Asian	2,010 (1.4)	2,418 (2.4)
Other/Unknown	2,455 (1.7)	2,537 (2.5)
T stage, n (%)		
T1-T2a	138,097 (93.6)	97,538 (95.6)
T2b-T2c	8,569 (5.8)	3,961 (3.9)
T3-T4	907 (0.6)	513 (0.5)
Insurance, n (%)		
Uninsured	1,498 (1.0)	1,812 (1.8)
Private Insurance	78,382 (53.1)	60,058 (58.9)
Medicaid	3,487 (2.4)	3,530 (3.5)
Medicare	59,293 (40.2)	33,225 (32.6)
Other Government	3,240 (2.2)	2,100 (2.1)
Missing	1,673 (1.1)	1,287 (1.3)

Facility location, n (%)		
New England	9,372 (6.4)	6,541 (6.4)
Middle Atlantic	15,757 (10.7)	24,755 (24.3)
South Atlantic	33,454 (22.7)	20,752 (20.3)
East North Central	28,214 (19.1)	18,195 (17.8)
East South Central	14,165 (9.6)	5,015 (4.9)
West North Central	14,327 (9.7)	7,877 (7.7)
West South Central	8,037 (5.5)	6,157 (6.0)
Mountain	7,345 (5.0)	2,500 (2.5)
Pacific	16,902 (11.5)	10,220 (10.0)

All p values <0.001 between the two treating facility types.
PSA= Prostate-specific antigen

Supplementary Table 2. Variation in prostate cancer treatment choice for localized Gleason Grade 1 prostate cancer by treating facility type.

	2010	2011	2012	2013	2014	2015	2016	2017	2018	2019
**Academic**										
Total, n	13017	13738	10303	9801	8718	9326	9549	9725	9216	8619
Active surveillance, n (%)	1573 (12.1)	2249 (16.4)	2268 (22)	2783 (28.4)	2950 (33.8)	3687 (39.5)	4403 (46.1)	4996 (51.4)	5186 (56.3)	5328 (61.8)
Radical prostatectomy, n (%)	7955 (61.1)	8103 (58.9)	5542 (53.8)	4828 (49.3)	4116 (47.2)	3958 (42.4)	3494 (36.6)	3255 (33.5)	2653 (28.8)	2176 (25.3)
Radiation therapy, n (%)	3279 (25.2)	3202 (23.3)	2354 (22.9)	2031 (20.7)	1524 (17.5)	1534 (16.5)	1526 (15.9)	1350 (13.9)	1279 (13.9)	1035 (12)
Androgen deprivation therapy, n (%)	99 (0.76)	101 (0.74)	72 (0.70)	75 (0.77)	53 (0.61)	86 (0.92)	69 (0.72)	67 (0.69)	47 (0.51)	40 (0.46)
Local ablation therapy, n (%)	111 (0.85)	83 (0.60)	67 (0.65)	84 (0.86)	75 (0.86)	61 (0.65)	57 (0.60)	57 (0.59)	51 (0.55)	40 (0.46)
**Non-academic**										
Total, n	20768	20614	14857	13784	12478	12521	13031	13342	12929	13249
Active surveillance, n (%)	1118 (5.4)	1561 (7.6)	1552 (10.5)	2120 (15.4)	2247 (18)	2920 (23.3)	3905 (29.9)	4932 (36.9)	5496 (42.5)	6439 (48.6)
Radical prostatectomy, n (%)	11132 (53.6)	11274 (54.7)	7837 (52.8)	6830 (49.6)	6099 (48.9)	5640 (45)	5350 (41)	4951 (37.1)	4219 (32.6)	3710 (28)
Radiation therapy, n (%)	7849 (37.8)	7171 (34.8)	5059 (34.1)	4447 (32.3)	3825 (30.7)	3700 (29.6)	3508 (26.9)	3191 (23.9)	2984 (23.1)	2906 (21.9)
Androgen deprivation therapy, n (%)	272 (1.31)	223 (1.08)	188 (1.27)	188 (1.36)	147 (1.18)	140 (1.12)	147 (1.13)	155 (1.16)	122 (0.94)	111 (0.84)
Local ablation therapy, n (%)	397 (1.91)	385 (1.87)	221 (1.49)	199 (1.44)	160 (1.28)	121 (0.97)	121 (0.93)	113 (0.85)	108 (0.84)	83 (0.63)

Supplementary Table 3. Average annual percent change for management strategies for Grade Group 1 (GG1) Prostate cancer by facility type

	**Academic**		**Non-academic**		**Difference**	
	AAPC [95%CI]	p-value	AAPC [95%CI]	p-value	AAPC Difference [95%CI]	p-value
Active surveillance	20.5 [18.6, 22.4]	<0.001	27.8 [24.1, 31.6]	<0.001	7.3 [3.6, 11.0]	≤0.01
Radical prostatectomy	-9.3 [-10.2, -8.5]	<0.001	-6.9 [-8.7, -5.2]	<0.001	2.4 [0.7, 4.1]	≤0.01
Radiation therapy	-7.9 [-8.8, -7.0]	<0.001	-5.9[-6.6, -5.3)	<0.001	2.0 [1.0, 2.9]	≤0.01
Androgen deprivation therapy	-4.1[-8.1,0.1]	0.053	-3.6 [-6.1, -1.0]	0.013	0.6 [-3.6, 4.7]	0.791
Local Ablation	-4.7[-8.5, -0.8]	<0.001	-11.4 [-13.0, -9.8]	0.025	-6.7 [-10.2, -3.1]	≤0.01

Supplementary Table 4. Variation in Grade Group 1 (GG1) Prostate cancer diagnoses by facility type

	**Non-academic centers**		**Academic centers**		**Unknown**	**Total**
	GG1 PCa cases	Total PCa cases	GG1 PCa cases	Total PCa cases		
Patients, n	147,573	709017	102,012	448954	601	1158572
Year of diagnosis, n (%)						
2010	20768 (28.4)	73204	13017 (30.1)	43245	76	116525
2011	20614 (27.7)	74502	13738 (30.1)	45688	90	120280
2012	14857 (23.9)	62125	10303 (26.1)	39446	58	101629
2013	13784 (22.4)	61434	9801 (24.8)	39536	71	101041
2014	12478 (20.8)	60119	8718 (22.8)	38283	49	98451
2015	12521 (19.1)	65536	9326 (21.7)	43013	58	108607
2016	13031 (18.7)	69787	9549 (20.8)	45875	45	115707
2017	13342 (17.5)	76413	9725 (19.5)	49996	60	126469
2018	12929 (15.8)	81606	9216 (17.4)	53042	46	134694
2019	13249 (15.7)	84291	8619 (17)	50830	48	135169

Supplementary Figure 1.

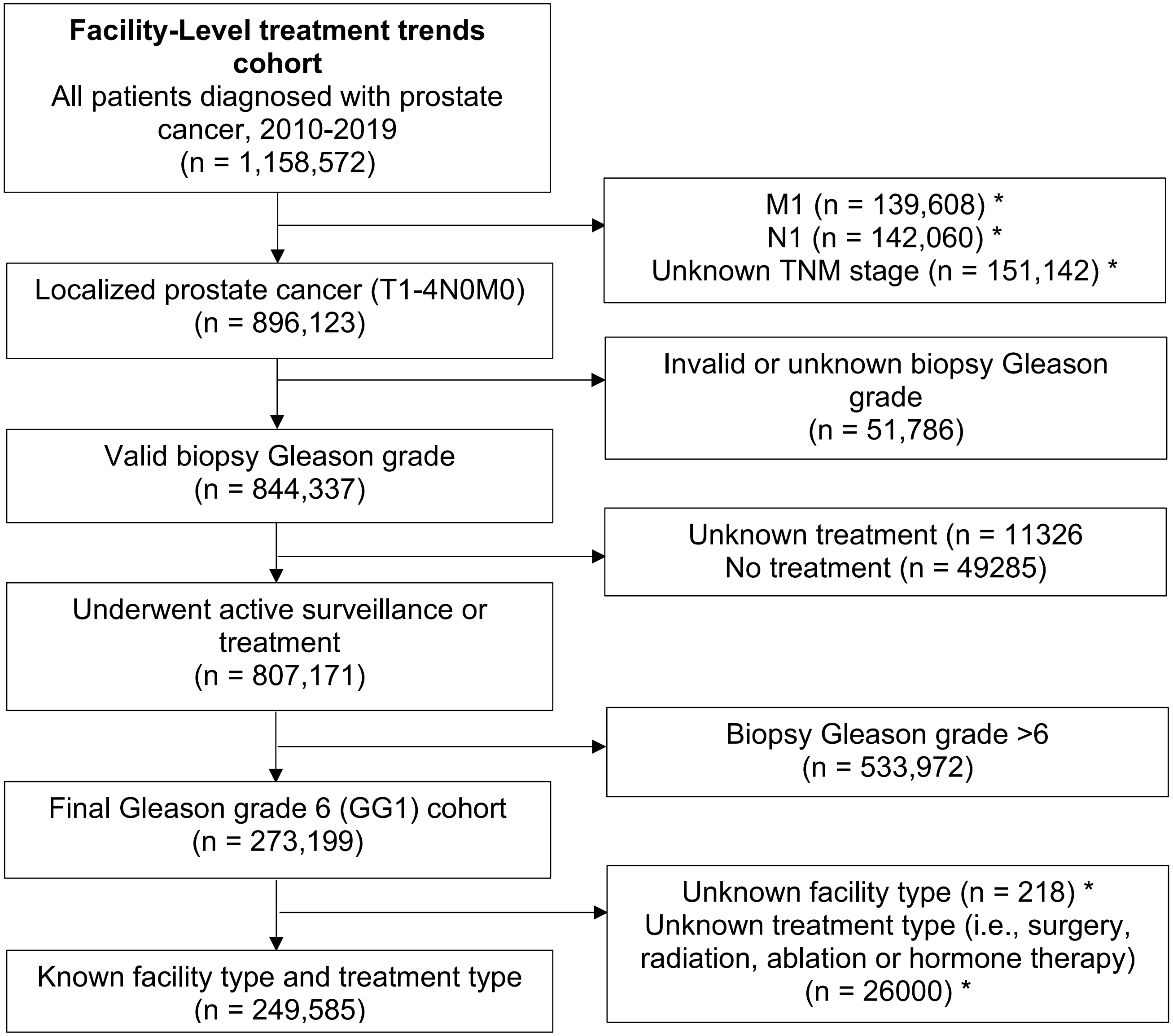

- (*) These levels were not mutually exclusive.

Supplementary Figure 2.

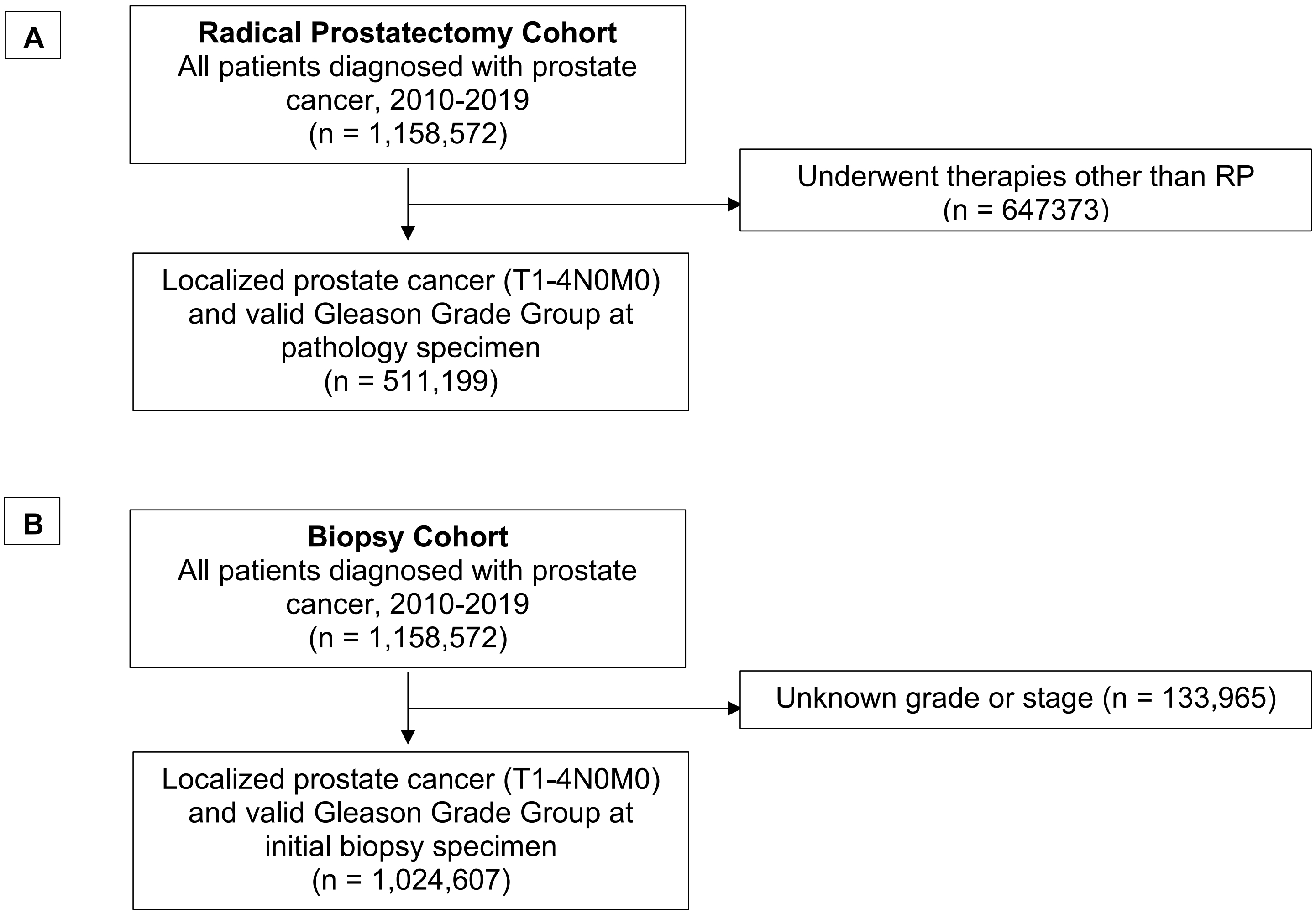

Supplement: pkad018_Supplementary_Data [file pkad018_supplementary_data.pdf]
